# A Deep Multi-Label Segmentation Network For Eosinophilic Esophagitis Whole Slide Biopsy Diagnostics

**DOI:** 10.1109/EMBC48229.2022.9871086

**Published:** 2022-07

**Authors:** Nati Daniel, Ariel Larey, Eliel Aknin, Garrett A. Osswald, Julie M. Caldwell, Mark Rochman, Margaret H. Collins, Guang-Yu Yang, Nicoleta C. Arva, Kelley E. Capocelli, Marc E. Rothenberg, Yonatan Savir

**Affiliations:** 1 Dept. of Physiology, Biophysics and System Biology, Faculty of Medicine, Technion, Israel.; 2 Faculty of Computer Science, Technion, Israel.; 3 Faculty of Industrial Engineering, Technion, Israel.; 4 Division of Allergy and Immunology, Cincinnati Children’s Hospital Medical Center, Dept. of Pediatrics, University of Cincinnati College of Medicine, OH, USA.,; 5 Dept. of Pathology, Cincinnati Children’s Hospital Medical Center, University of Cincinnati College of Medicine, Cincinnati, OH, USA.; 6 Dept. of Pathology, Ann & Robert H. Lurie Children’s Hospital of Chicago, Northwestern University, The Feinberg School of Medicine, Chicago, IL, USA.; 7 Dept. of Pathology, Children’s Hospital Colorado, Aurora, CO, USA.

**Keywords:** Decision support system, deep learning, digital pathology, eosinophilic esophagitis, whole slide image segmentation and classification

## Abstract

Eosinophilic esophagitis (EoE) is an allergic inflammatory condition of the esophagus associated with elevated numbers of eosinophils. Disease diagnosis and monitoring require determining the concentration of eosinophils in esophageal biopsies, a time-consuming, tedious and somewhat subjective task currently performed by pathologists. Here, we developed a machine learning pipeline to identify, quantitate and diagnose EoE patients’ at the whole slide image level. We propose a platform that combines multi-label segmentation deep network decision support system with dynamics convolution that is able to process whole biopsy slide. Our network is able to segment both intact and not-intact eosinophils with a mean intersection over union (mIoU) of 0.93. This segmentation enables the local quantification of intact eosinophils with a mean absolute error of 0.611 eosinophils. We examined a cohort of 1066 whole slide images from 400 patients derived from multiple institutions. Using this set, our model achieved a global accuracy of 94.75%, sensitivity of 94.13%, and specificity of 95.25% in reporting EoE disease activity. Our work provides state-of-the-art performances on the largest EoE cohort to date, and successfully addresses two of the main challenges in EoE diagnostics and digital pathology, the need to detect several types of small features simultaneously, and the ability to analyze whole slides efficiently. Our results pave the way for an automated diagnosis of EoE and can be utilized for other conditions with similar challenges.

## I. INTRODUCTION

Eosinophilic esophagitis (EoE) is a chronic allergic inflammatory condition of the esophagus characterized by elevated levels of esophageal eosinophils [1]. After gastroesophageal reflux disease, EoE is the most common cause of chronic esophagitis leading to symptoms such as dysphagia and esophageal food impaction [2]. Food hypersensitivity, allergic inflammation, and multiple genetic and environmental factors are the main drivers of the disease pathogenesis [3]. The diagnosis of EoE requires a manual microscopic review of endoscopic biopsies, and the diagnostic threshold of at least 15 eosinophils/400X high-power field (HPF) is required. Hematoxylin and eosin (H&E) staining is used frequently to detect eosinophilic cells, as their unique basically charged granule constituents have an affinity for the eosin stain [4]. A common practice is to identify the area of tissue within a slide that exhibits the densest eosinophil infiltration and quantify the peak eosinophil count (PEC) in that particular HPF and compare it to a predetermined threshold [4]–[6]. It is crucial to distinguish between intact eosinophils, which have both their intensely red cytoplasmic granules and nucleus visible [6] and contribute to the PEC, and not-intact eosinophils without granules or visible nucleus that are not added to the PEC.

Detecting and counting different cellular features is a laborious and time-consuming task that leads to inconsistencies even between trained observers [4]–[6]. The field still lacks a robust automatic process that can cope with the task of counting inflammatory cells such as eosinophils and aid the pathologists. Therefore, machine and deep learning techniques were utilized for various tasks in digital pathology [7], [8]. For example, identify types of cancerous lesions [9], [10], to segment cell nuclei [11], to segment inflammatory bowel disease (IBD) tissue features [12], to classify different cancer types via histology images [13], [14], to perform cancer screening [15], and to personalize cancer care [16]. Yet, several fundamental challenges still remain, particularly the gap between the scale of the features that determine the medical condition (which can be in the scale of a few cells) and the overall content of an entire slide that has a typical size of 10^8^ − 10^10^ pixels (much larger than the typical input size of most architectures which is about 10^6^ pixels).

One approach for predicting the patient outcome is to train deep networks based on one global label for each slide (e.g., whether a patient has active EoE or not) and train the network without local semantic labeling the image. The main advantage of this approach is that it does not require intensive labeling effort and allows the machine to infer representations of a condition without a priori local bias. We have recently used this approach to develop a deep learning system for classifying H&E-stained esophageal tissue images based on global labeling [17]. Using this approach led to a classification of EoE disease activity with an accuracy of approximately 85% based only on global labels. Furthermore, these results showed that histological features associated with EoE are not only local clusters of eosinophils but also global attributes of the histology pattern.

A second approach for outcome prediction is to use semantic segmentation. In this case, each pixel in the image is labeled according to its tissue and cellular type, and the network is then trained to identify local regions belonging to the same classes in separate images. In this approach, the classification of the patients depends on spatial scores such as the area or number of particular features. One of the challenges of this approach is that in many cases, such as EoE, there are features which are very similar but have different clinical implications. In the case of EoE, it is critical to distinguish between eosinophils that are intact and ones that are non-intact. These cells look very similar but only the intact ones contribute to the PEC score.

Various net architectures have been developed for segmentation, such as Mask RCNN [18], Mask ECNN [19], DeepLab [20], [21], and Generative Adversarial Networks [22]. U-Net [23] is a common architecture model for biomedical image segmentation that was previously used for segmenting many cell types, including eosinophils [24]. This network is based on an auto-encoder architecture in which the encoder takes the input, performs down-sampling, and outputs a feature vector/map that represents the input. The decoder does the opposite orientation (i.e., up-sampling) that takes the feature vector (i.e., the features) from the encoder and gives the best closest match to the actual input or intended output. An upgraded model of the U-Net, called UNet++ [25], introduced re-designed skip pathways between the original U-Net layers. These pathways aim to reduce the semantic gap between the feature maps of the encoder and decoder sub-networks. Another update for UNet++ is the deep supervision that combines different sub-models of the full UNet++ to segment the image and enables more accurate segmentation, particularly for lesions that appear at multiple scales.

Here, we develop an efficient dynamic convolution pipeline, on the one hand, allows our proposed model to run rapidly on whole slide image in term of run time and memory constraints, and on the other hand, to segment a multi-label classes of intact and not-intact eosinophils for EoE diagnostics in high accuracy. We test our pipeline on the largest EoE cohort to date, and show that we achieved excellent segmentation performances with a mean intersection over union metric (mIoU) score of 0.93, allowing state-of-the-art eosinophils counting with a standard error of 0.611. Furthermore, based on the eosinophil counts, the model can classify EoE disease local activity according to whether the PEC is greater than 15/HPF with an accuracy of 98.48%, a sensitivity of 96.89%, and specificity of 98.89%. Finally, we validated our model on the whole slide level using a cohort of 1066 biopsy slides from 400 patients and achieved an accuracy of 94.75%, sensitivity of 94.13%, and specificity of 95.25%.

## II. METHODS

### A. Study population and datasets

This study was conducted within the context of the Consortium of Eosinophilic Gastrointestinal Disease Researchers (CEGIR, https://www1.rarediseasesnetwork.org/cms/cegir) [26], a national collaborative network of 16 academic centers caring for adults and children with eosinophilic gastrointestinal disorders. The CEGIR clinical trial, Outcomes Measures in Eosinophilic Gastrointestinal Disorders across the Ages (OMEGA), is a longitudinal cohort study aimed at understanding the natural history of EoE, eosinophilic gastritis, and eosinophilic colitis during routine clinical care. All subjects’ clinical data were stored at the Data Management and Coordinating Center (DMCC) at Cincinnati Children’s Hospital Medical Center. Data were systematically extracted from the databases. This study was approved by the institutional review boards of the participating institutions via a central institutional review board at Cincinnati Children’s Hospital Medical Center (CCHMC IRB protocol 2015-3613). Participants provided written informed consent. 419 subjects undergoing endoscopy (EGD) for standard-of-care purposes agreed to have their clinical, histologic, and demographic information stored in a private research database. Distal, mid, or proximal esophageal biopsies (1–3 per anatomical site) per patient were placed in 10% formalin; the tissue was then processed and embedded in paraffin. Sections (4*μm*) were mounted on glass slides and subjected to hematoxylin and eosin (H&E) staining. Slides were scanned on the Aperio scanner at 400X magnification and were saved in SVS format. Each slide of esophageal tissue was analyzed by an anatomic pathologist who is a member of the CEGIR central pathology core to determine peak eosinophil count per 400X high-power field (HPF). The peak eosinophil counts associated with each image were stored at the DMCC.

### B. Semantic labeling

WSI images from 23 biopsy slides from 19 patients were used for semantic labeling. These had a median size of about 150K × 56K pixels. These images were split into patches with a size of 1200×1200 pixels. Patches contained less than 15% background were filtered to balance edges frequency. The remaining patches (n = 10,170) were annotated by a trained, experienced researcher, and were validated by four experts. Each pixel was assigned to one of three classes: intact eosinophils, defined by eosinophils with visible intensely red cytoplasmic granules and nucleus; not intact eosinophils, defined by eosinophils without a visible nucleus or large groups of extracellular eosinophil granules (Table I); or non-eosinophils. The annotator marked the center of the intact or not intact eosinophils and a circle with a diameter of 50 pixels was used to generate the mask. We used this dataset to train the multi-label deep semantic segmentation network.

### C. Segmentation metrics

To estimate the segmentation performances, we used the following metrics,

(1) $$\text{mIoU}=\frac{1}{I\cdot C}\underset{i}{\sum }\underset{c}{\sum }\frac{TP_{i,c}}{TP_{i,c}+FP_{i,c}+FN_{i,c}}$$

(2) $$\text{mPrecision}=\frac{1}{I\cdot C}\underset{i}{\sum }\underset{c}{\sum }\frac{TP_{i,c}}{TP_{i,c}+FP_{i,c}}$$

(3) $$\text{mRecall}=\frac{1}{I\cdot C}\underset{i}{\sum }\underset{c}{\sum }\frac{TP_{i,c}}{TP_{i,c}+FN_{i,c}}$$

(4) $$\text{mSpecification}=\frac{1}{I\cdot C}\underset{i}{\sum }\underset{c}{\sum }\frac{TN_{i,c}}{TN_{i,c}+FP_{i,c}}$$

where the *c* index iterates over the different classes in the image, and the *i* index iterates over the different images in the dataset. *C* is the total number of classes, and *I* is the total number of images. *TP*, *TN*, *FP*, *FN* are classification elements that denote true positive, true negative, false positive, and false negative of the areas of each image, respectively. mIoU is the intersection between the actual and predicted areas divided by their union, mPrecision is the fraction of the true positive out of the total predicated area, mRecall is the fraction of the true positive out of the total ground truth area being positive, and mSpecification is the fraction of the true negative out of the total ground truth area being negative.

### D. Training procedure

The labeled images were split arbitrarily into training (80%) and validation (20%) sets. Using a rectangular grid, each image was cropped into nine 448×448 pixel patches. These patches were then used as an input for a model network based on the implementation of UNet++ (which achieved better performance than U-Net). The updated model was trained and optimized using Pytorch [27] framework on a single NVIDIA GeForce RTX 2080 Ti GPU. During the training, different hyperparameters were examined. The “Cosine Annealing” learning rate scheduler, patch size of 448×448 pixels with a stride of 376×376 pixels size, batch size of 5, 100 epochs, and 0.5 softmax threshold were revealed to be optimal. Moreover, the optimal loss function includes a Dice metric and a binary cross-entropy (BCE) element, where the Dice and BCE are weighted with values of 1 and 0.5, respectively. The output of the model network was converted into a binary segmentation mask using a 0.5 probability threshold. The masks of the different patches were combined to reconstitute the original 1200×1200 image using OR function (i.e., is the truth-functional operator of inclusive disjunction) for the overlapping regions. During the training process, we examined different models based on eight different segmentation metrics, including mIoU, mPrecision, mRecall, and weighted average combinations between the Precision and Recall.

### E. Estimating the eosinophils numbers and density

After semantic segmentation, the average area of a typical eosinophil is 2050 pixels. Each connected region with an area larger than 1800 and less than or equal to 3000 pixels was counted as one eosinophil. In a connected region that is larger than 3000 pixels, each additional 2000 pixels adds one eosinophil to the eosinophil count of this connected region. Based on this method, we calculate the intact eosinophil density for each image.

### F. Classifying active and not-active images

After calculating the intact eosinophil density, we compare it to the cutoff density used by pathologists of 15 intact eosinophils per 400X HPF (area of 0.3*mm*^2^, which corresponds to a size of 2144×2144 pixels).

### G. Pipeline Architecture

Our pipeline input can be either one EoE patient’s whole slide image (WSI) or several patients’ WSIs, whereas the output includes three main elements:

1. Segmented WSIs. Each pixel of the WSIs is labeled as belonging to the relevant class (i.e., intact eosinophils, not intact eosinophils, or non-eosinophils). This output would aid the pathologist’s search for important regions.
2. PEC. The peak eosinophil count represents the intact intraepithelial eosinophil count of the most densely infiltrated high-power field for each WSI.
3. A ranked eosinophil count list. It can help the pathologist prioritize between severe to non-severe patients.

**Fig. 1.**
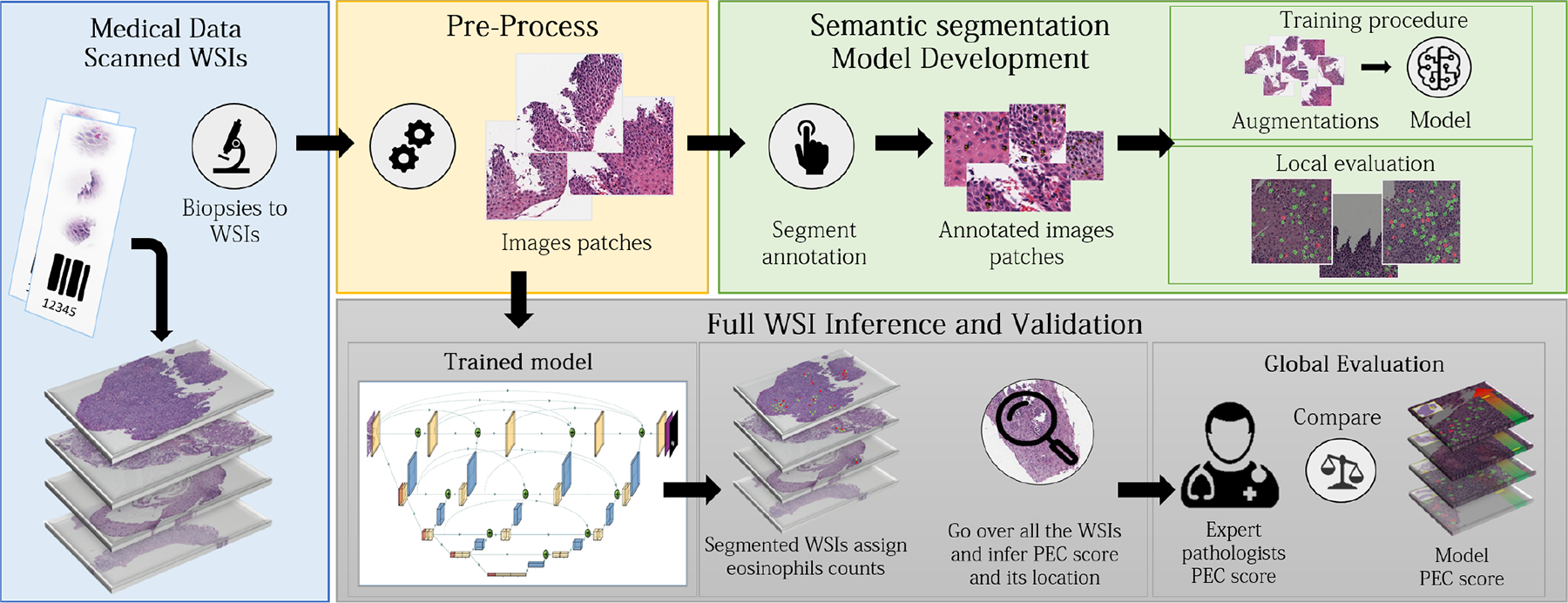
Overview of network training and procedure. The model network high-level architecture is presented. For training, whole slide images (WSIs) were pre-processed and cropped into patches on which a trained researcher marked the location of both intact and not intact eosinophils. The trained multi-label semantic segmentation network can get a WSI as an input, segment it, count the two types of eosinophils, locate the area with the highest number of eosinophils, count its value, and calculate the peak eosinophil count per high power field (PEC score). The model network was validated using 1066 whole slide images from 400 patients for which the PEC had been previously determined by pathologists.

For inference, the WSI’s global PEC is calculated by counting intact eosinophils using a kernel in the size of the HPF of an area of 0.3*mm*^2^, and iterating over the slide with a stride of 500×500 pixels to find the maximum eosinophil count within the HPF boundaries, which corresponds to a size of 2144×2144 pixels (548*μm×*548*μm*). Each HPF is cropped into patches of 448×448 pixels size (1792*μm×*1792*μm*), with a stride of 424×424 pixels, in order to be adjusted to the deep learning net input size. Then, every patch is fed into the model network and segmented. Finally, all the segmentation patches masks (a typical number of 10K–100K patches) are fused back to the full size of the original WSI, and a full segmented EoE patient’s WSI is produced. This procedure was done using dynamic convolution, which allows the model network to run rapidly within the processing speed, and enables scalability in WSI size in terms of memory constraints of a standard computer. This technique involves the process of obtaining and analyzing only the relevant patches for each HPF that corresponds to a specific dynamic convolution iteration. The overall flow is described in Fig. 1.

## III. RESULTS

### A. Semantic Segmentation

**Fig. 2.**
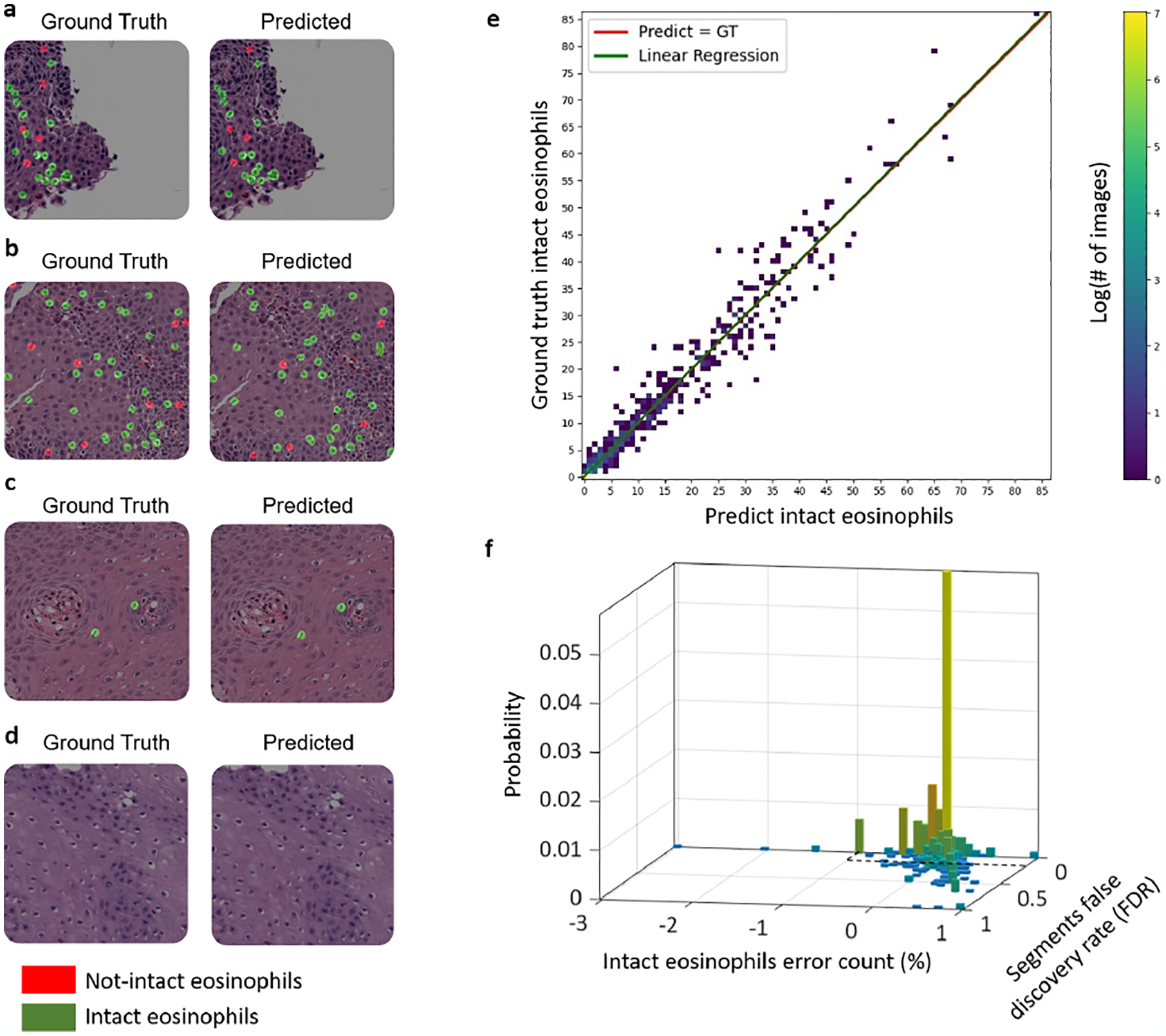
Quality of multi-label segmentation. Examples of multi-label segmentation (a–d). The size of each patch is 1200×1200 pixels. The left images are colored with the ground truth mask’s features, and the right replication is colored with its corresponding prediction mask’s features. The intact Eos class is colored in green, and the not-intact Eos class is colored in red. (a) and (b) are examples of slides from EoE patients with many eosinophils per patch/HPF, (c) is an example of an EoE patient with only two eosinophils at the local level per patch, and (d) is an example of an EoE patient without any eosinophils at the local level per patch. (e) The relationship between the true number of eosinophils and the number estimated from semantic segmentation of a 1200×1200 patch. The best linear fit has a slope of 1.005 (95% confidence interval values of 0.963 and 1.013), an intersection of 0.03 (confidence interval values of −0.065 and 0.126), and an R-square of 0.9721. The mean absolute counting error is 0.611 eosinophils. (f) In 90% of the images, both the connected segments’ false discovery rate is smaller than 0.1, and the counting error rate is lower than 3% (the area defined by the black dashed lines).

We first validated the ability of the UNet++ model to detect and segment both intact and not-intact eosinophils. The network segments them with mIoU of 93%, mPrecision of 95% for intact eosinophils and 97% for not intact eosinophils, and mRecall of 97% for intact eosinophils and 95% for not-intact eosinophils. In addition, we trained another segmentation model based on U-Net and compared its segmentation metrics scores to the scores achieved by UNet++ (Table II). Since UNet++ is superior to UNet with respect to all segmentation metrics, we implemented our model based on UNet++. We also evaluated the ability of our network model to distinguish between the two types of eosinophils (Fig. 2a–d). Out of the true intact eosinophils, 98.8% were identified correctly as intact. This performance is critical to the ability of the model network to provide a reliable intact eosinophil count.

### B. Optimizing counting of intact eosinophils and image classification

Next, we further optimized the network for the tasks of counting intact eosinophils and classification according to eosinophils density. The original UNet++ optimization procedure calculates the mIoU per batch, and uses it as the optimization metric. As our data, as many histological datasets that involve rare single-cell features, contains many images without eosinophils, optimizing over a batch metric can lead to a bias. Moreover, there is a tradeoff between high precision, underestimating the number of eosinophils, and high recall and overestimating the number of eosinophils. To account for these factors, we trained eight models using eight different metrics per image (rather than per batch). Table III summarizes the performances of each model in terms of quantification and classification, according to eosinophils density performances. The model that provides the best results for both counting and classification is the one in which the optimization metric was the arithmetic mean of recall and precision. This model has a mean counting error of 0.611, classification accuracy of 98.48% and F1-score of 96.30%. Interestingly, the metric that provided the second best counting error was a weighted average of 0.4 recall and 0.6 precision. This metric counting error was much better than 0.6 precision and 0.4 recall case and better than the mIoU. This suggests that avoiding underestimating eosinophils numbers has a bigger weight than avoiding overestimating eosinophils numbers during the training process.

Fig. 2e–f illustrates the counting performances. The mean count error is 0.611 eosinophils. The best linear fit for the relationship between the predicted counts and ground truth has a slope of 1.005 (95% confidence interval values of 0.963 and 1.013) and an intersection of 0.03 (confidence interval values of −0.065 and 0.126), R-square of 0.9721 and F-test p-value ≪ 0.001 (Fig. 2e). That is, we observed no inherent bias in the counting estimation. Another measure for the quality of the segmentation and counting is that both the counting error and the actual false discovery rate of the labeled masks are low and correlated (Fig. 2f). In 90% of the images, the connected segment’s false discovery rate is smaller than 0.1, and the counting error rate is lower than 3%. As such, the correct counting is indeed the result of correct local detection.

### C. Network Validation on the Whole slide image level

**Fig. 3.**
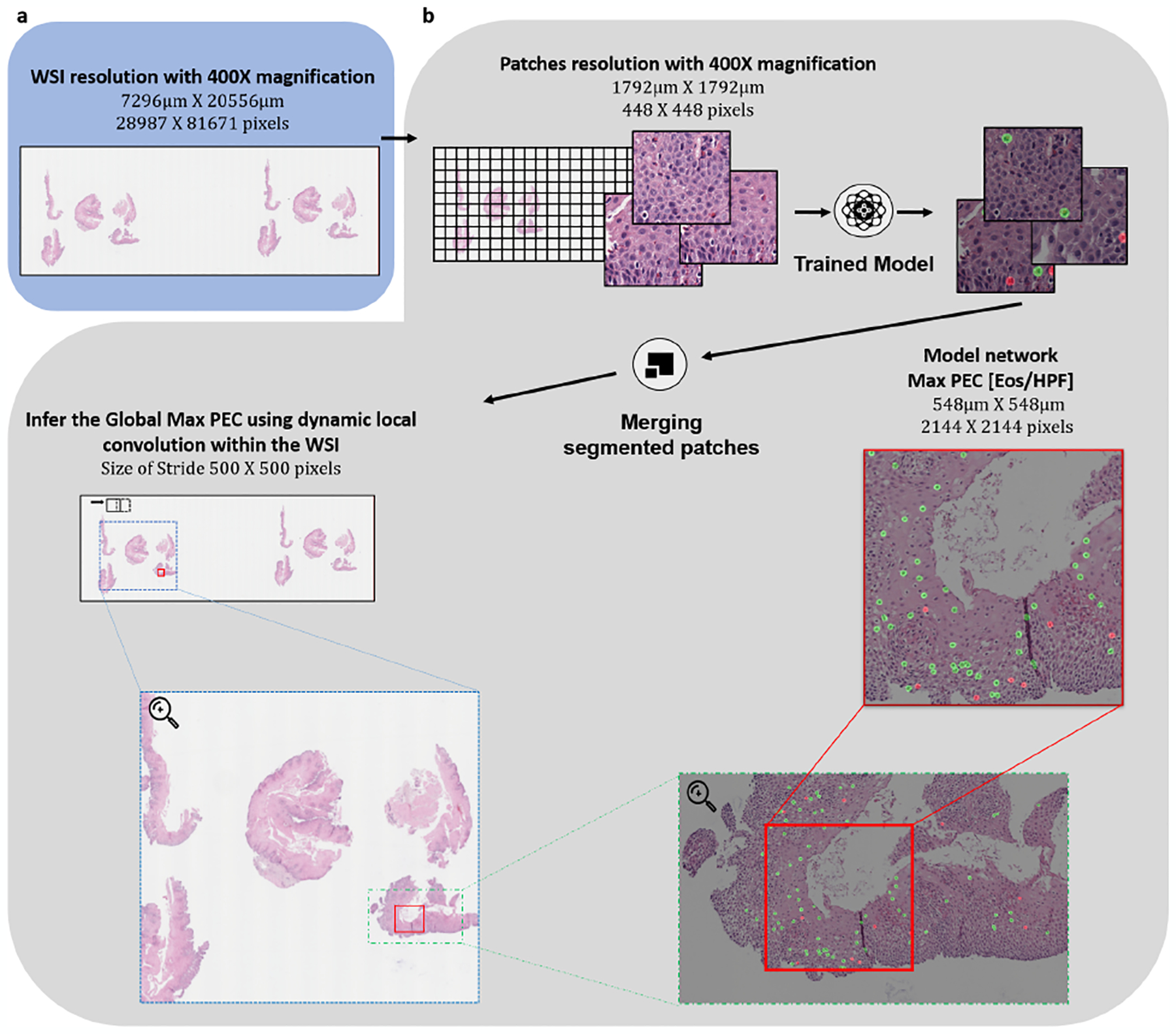
Applying our pipeline to whole slide images (WSIs). The figure presents an example of an analysis of a specific WSI in the different scales. The red box denotes the location of the HPF where the Network’s maximum PEC was found.

To validate the ability of the network to estimate the peak eosinophil count of a whole slide, we examined a cohort of 1066 biopsy slides from 400 patients. These biopsy slides differ from the 23 slides used for training the network. In this cohort, each WSI was derived from a scan done at 400X magnification, resulting in input images with a width of 20K–100K pixels and a typical length of 100K–200K pixels. An example of a WSI’s prediction process is presented in Fig. 3. These slides were scored by a pathologist who examined the slides and estimated the PEC per slide, that is, the maximal intraepithelial eosinophil count observed in an HPF of 0.3*mm*^2^, which corresponds to a size of 2144×2144 pixels.

**Fig. 4.**
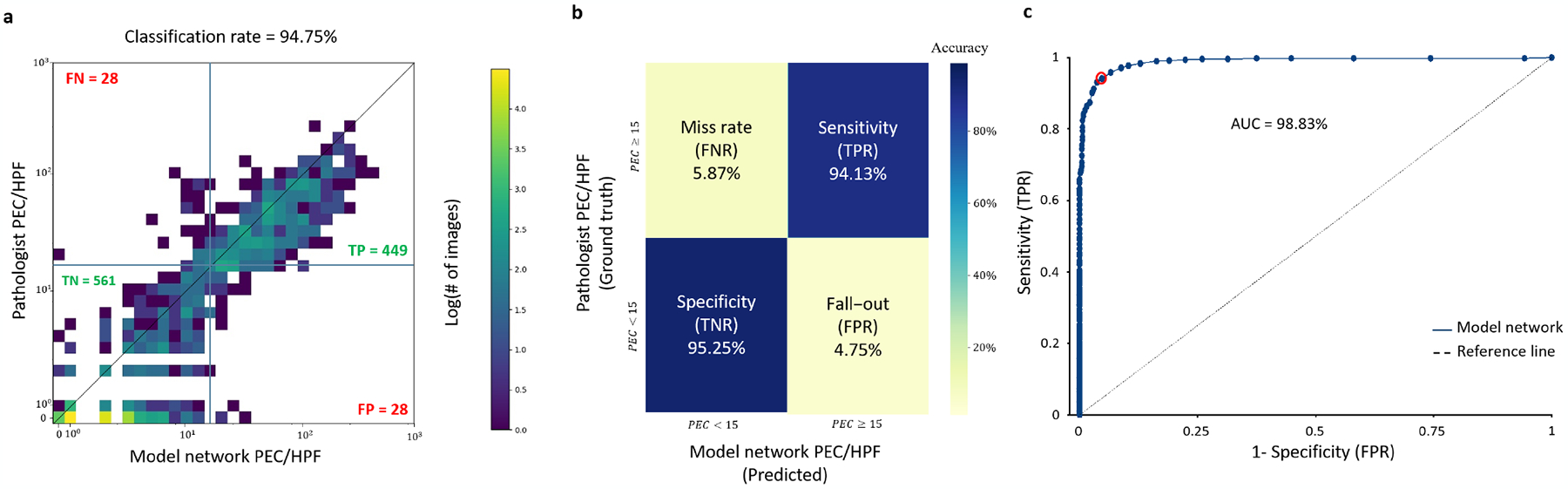
Network validation on a cohort of 1066 slides from 400 EoE patients. (a) Comparing the maximal intact eosinophils per HPF (0.3*mm*^2^) of an entire slide, as measured by the pathologists and the model network. (b) Classification confusion matrix based on comparing the peak eosinophil count in a high-power field to 15. (c) The classifier performance in the AUC space; each dot represents a different PEC threshold. A threshold of 14.5 (red circle) maximizes the overall accuracy.

The clinical diagnostic threshold density for classifying patients with active EoE is a count of greater than or equal to 15 eosinophils in at least one HPF. Fig. 4 shows the comparison between the model network count and the pathologist’s count, and the resulting classification. Note that in this case, the algorithm identifies the HPF with the highest number of eosinophils without prior knowledge of the specific HPF chosen by the pathologist. Our pipeline provides an accuracy of 94.75% with a sensitivity of 94.13% and specificity of 95.25% (Fig. 4a, b). The area under the curve is 98.83%, and the critical threshold that maximizes the accuracy is 14.5 (Fig. 4c). The classification accuracy for defining PEC in the biopsies obtained from different spatial locations, proximal, mid, and distal esophagus, were 96.2%, 92.9%, and 93.3%, respectively.

## IV. DISCUSSION

The digital transformation of pathology is expected to grow dramatically over the next few years as increasing numbers of laboratories gain access to high throughput scanning devices and aim to automate the analysis of scanned microscopic images. This transformation is driven by several factors, including the prolonged time per case due to the growing complexity of pathological criteria for diagnosing and monitoring diseases compounded by the limited supply of pathologists, especially in different geographical regions.

There are inherent challenges in digital pathology beyond data collection. One of the main challenges is the large textual variation of slides and the existence of multiple length scales – slides that are very large compared with the features that define the clinical condition. Moreover, the typical size of a slide is much larger than the typical image input size of current convolutional networks. Thus, even if a label exists at the slide level, training is challenging. On the other hand, labeling at the pixel level is laborious, particularly if there are multiple tissue features relevant to the clinical condition.

Herein, we developed a decision support system that is trained to identify two classes of eosinophils simultaneously and included an efficient dynamic convolution technique that scans the local segmented EoE features. This multi-label approach is different from training two different networks to identify each feature individually and relieve the problem of conflicting overlap regions.

EoE is an example of a condition that relies on the identification and counting of small objects, such as single cells, within a whole slide. The pathologist is required to scan the slide and evaluate the local concentration of eosinophils. The typical region that is used for counting is the size of 0.3*mm*^2^, and thus it is laborious to probe the entire slide. The validation cohort we used in the study is the largest to date and includes 1066 biopsy slides from 400 patients. Each slide has a PEC score of the maximal eosinophil density as determined by a body of expert pathologists associated with CEGIR. This score is based on the pathologist’s conclusion and reflects only the number of high-power fields that the pathologist probed. Thus, a clear advantage of our pipeline is that every possible high-power field can be probed for each slide.

Our model provides state-of-the-art segmentation of eosinophils that can discriminate between intact and not-intact eosinophils (Table II). This segmentation results in an intact eosinophil count that differs from the human expert counts by a mean absolute error of 0.611 eosinophils on identical slides. The counting on the annotated dataset yields a classification accuracy of 98.48%. One of the main advantages of our dataset is that it allows testing the counting performances on a large dynamic range of eosinophils ranging from zero to hundreds. Thus, our counting performances are tested in a real-life scenario.

While the model network has excellent performances on the patch level, each patch’s area is three orders of magnitude smaller than the area of a whole slide image. Our pipeline uses a dynamics convolution approach to segment the entire slide rapidly and estimates the maximal eosinophil density of any given high power field size. This is an essential feature as different pathologists may use distinct sizes of a HPF, leading to ambiguity between pathologists. Using the model network allows the user to give the size of the HPF as an input. Comparing the model network PEC score (using a HPF size of 0.3*mm*^2^) and the score from the CEGIR dataset resulted in a remarkable agreement between the machine and the human score and classification (Fig. 4). Translating the score agreement to the decision-making depends on the threshold. When taking different thresholds and comparing the performance in the ROC space, the optimal accuracy is obtained with a threshold of 14.5/HPF; that is, for integer values, the best threshold is larger-equal than 15. This result highlights the lack of bias in model’s counting as this is the same threshold recommended by field experts [1].

Our work highlights the importance of multi-labeling capacity on small features and the ability to deploy the model network on a whole slide image rapidly and efficiently. These findings are a significant step toward an automated diagnosis of EoE using digital pathology and have implications for analyzing other biopsy-based disease diagnoses.

## Figures and Tables

**TABLE I:** Semantic labeling statistics at the 1200×1200 patches level.

Class	Number of images (percent of total)^a^	Total Area (pixels)^b^	Mean fraction out of total patch area^c^
Eos-not intact	2028 (19.94%)	24.37M	0.83%
Eos-intact	2317 (22.78%)	78.47M	2.35%

a, The total number and percentages of 1200×1200 pixel images containing at least one instance of the indicated class of eosinophil.

b, The sum of the area (in pixels) of the indicated class of eosinophil.

c, The average percentage of pixels classified as the indicated class per total patch area.

Eos, eosinophils; M, million.

**TABLE II:** Segmentation metrics scores on the validation set. For each metric, the highest overall score is marked in bold.

Metric	Model	Intact eosinophils	Not-intact eosinophils	Overall
mloU (1)	UNet++	0.93	0.93	**0.93**
	U-Net	0.84	0.8	0.82
mPrecision (2)	UNet++	0.95	0.97	**0.96**
	U-Net	0.87	0.89	0.88
mRecall (3)	UNet++	0.97	0.95	**0.96**
	U-Net	0.92	0.86	0.89
mSpecificity (4)	UNet++	0.998	0.999	**0.998**
	U-Net	0.997	0.999	**0.998**

IoU, intersection over union; m, mean.

**TABLE III:** Classification and quantification scores on the validation set. During the training process, eight different models were optimized based on different segmentation metrics. All models are ranked based on their scores positions for each metric individually (lower is better). Averaging the ranks over the classification metrics derives the Mean Rank for each model. The Final Rank is taken as the lower Mean Rank per model. Thus, the best quantification and classification ranks were achieved by the m(0.5P + 0.5R) model, and are marked in bold. This model was chosen as our network model.

Model	Intact eosinophils quantification	Classification metrics							
	Mean count error / Ra	Accuracy / Ra	F1-score / Ra	Miss-rate (FNR) / Ra	Fall-out (FPR) / Ra	Specificity (TNR) / Ra	Sensitivity (TPR) / Ra	Mean Ra	Final Ra
mIoU	0.669 / 3	98.42% / 2	96.09% / 2	4.66% / 5	0.79% / 4	99.21% / 4	95.34% / 5	3.67	2
mRecall	1.686 / 8	97.22% / 5	93.39% / 5	3.42% / 3	2.62% / 7	97.38% / 7	96.58% / 3	5	5
mPrecision	1.561 / 6	94.94% / 8	85.82% / 8	24.84% / 8	0% / 1	100% / 1	75.16% / 8	5.67	8
m(0.3P + 0.7R)	1.263 / 5	97.22% / 5	93.39% / 5	3.42% / 3	2.62% / 7	97.38% / 7	96.58% / 3	5	5
m(0.4P + 0.6R)	0.651 / 2	98.23% / 4	95.72% / 4	2.80% / 1	1.51% / 6	98.49% / 6	97.20% / 1	3.67	2
m(0.5P + 0.5R)	0.611 / **1**	98.48% / 1	96.30% / 1	3.11% / 2	1.11% / 5	98.89% / 5	96.89% / 2	2.67	**1**
m(0.6P + 0.4R)	0.907 / 4	98.42% / 2	96.04% / 3	5.90% / 6	0.48% / 3	99.52% / 3	94.10% / 6	3.83	4
m(0.7P + 0.3R)	1.561 / 6	96.33% / 7	90.10% / 7	18.01% / 7	0% / 1	100% / 1	81.99% / 7	5	5

Accuracy (number of images accurately classified as either active EoE (a count of greater than or equal to 15 eosinophils in at least one HPF) or not-active EoE (a count of lower than 15 eosinophils in at least one HPF) / total number of images × 100), F1-score (the harmonic mean of the precision and recall; formulated as = 2*/*(*Precision*^−1^ + *Recall*^−1^), Miss-rate (TNR; number of images classified incorrectly as not-active EoE / number of active EoE images × 100), Fall-out (FPR; number of images classified incorrectly as active EoE / number of not-active EoE images × 100), True positive rate (TPR; number of images classified as active EoE / number of active EoE images × 100), and true negative rate (TNR; number of images classified as not-active EoE / number of not-active EoE images × 100). IoU, intersection over union; m, mean; P, Precision; R, Recall; Ra, Rank.
